# Cost-Effective Inorganic Multilayer Film for High-Performance Daytime Radiative Cooling

**DOI:** 10.3390/ma18081729

**Published:** 2025-04-10

**Authors:** Huan Liu, Yingxin Yang, Atsha Ambar, Zhiqiang Fan, Ying Sun, Cong Wang

**Affiliations:** 1 School of Energy and Power Engineering, Beihang University, Beijing 100191, China; by2004511@buaa.edu.cn (H.L.); yyx150@buaa.edu.cn (Y.Y.); 2 School of Physics, Beihang University, Beijing 100191, China; atshaambar@buaa.edu.cn; 3 School of Integrated Circuit Science and Engineering, Beihang University, Beijing 100191, China; zhiqiangf@buaa.edu.cn; 4 National Key Laboratory of Spintronics, Hangzhou International Innovation Institute, Beihang University, Hangzhou 311115, China

**Keywords:** inorganic multilayer films, radiative cooling, interface adhesion, atmospheric window, cost-effective

## Abstract

Inorganic multilayer films for radiative cooling have garnered significant attention due to their exceptional resistance to photothermal degradation. However, the design and fabrication of structurally simple and cost-effective inorganic multilayer films remain challenging due to limitations in material properties and the preparation process. This study develops a structurally simple inorganic multilayer film (Si_3_N_4_/SiO_2_/Al_2_O_3_/Si_3_N_4_/Al) for daytime radiative cooling. Instead of the conventional periodic alternation of high and low refractive indices (H-L…H-L), this work proposes a H-L-L-H symmetric multilayer film structure to achieve improved radiative cooling performance. The fabricated multilayer film demonstrates superior radiative cooling properties and lower thickness than that in the current studies using Al as the reflective layer, achieving a solar reflectance of 89.57%, an atmospheric window (8–13 μm) emissivity of 83.41%, and a net cooling power of 63.38 W·m^−2^. Under direct sunlight, the multilayer film demonstrated a maximum temperature reduction of approximately 3 °C compared to the reference sample. By employing a thermal treatment process for the Si_3_N_4_ layer, the poor adhesion between the Al layer and the Si_3_N_4_ layer is successfully addressed without compromising optical performance. The underlying physical mechanisms are also elucidated. This work provides an effective strategy for developing daytime radiative cooling inorganic multilayer films suitable for large-scale industrial production.

## 1. Introduction

Radiative cooling [1,2,3,4], as a passive cooling mechanism, enables heat dissipation without energy consumption and has been extensively applied in spacecraft thermal management, electronic device cooling, building temperature regulation, and water harvesting in arid regions [5,6,7,8,9]. For effective daytime radiative cooling within the atmosphere, materials must simultaneously demonstrate two essential characteristics: high emissivity within the atmospheric window (8–13 μm) and high reflectivity in the solar spectrum region (0.3–2.5 μm). The strategic utilization of these properties enables significant daytime radiative cooling performance [7,10,11]. Recent developments have witnessed the establishment of several companies dedicated to commercializing radiative cooling materials. Commercial radiative cooling structures require not only exceptional stability but also simple structures and low production costs [12]. In previous studies, it was a great challenge to realize both cost-effective and high performance in radiative cooling multilayers. Consequently, there is an urgent need in the radiative cooling field for material systems that combine simple structures, low production costs, excellent radiative cooling performance, and enhanced suitability for commercial applications [13,14,15].

Recent advancements in radiative cooling have focused on developing materials and structures that optimize both solar reflectance and infrared radiation [16,17,18]. However, most of them rely on expensive materials or complex film structures, limiting their scalability and practical application. Inorganic materials, known for their durability, thermal stability, and cost-effectiveness, offer a viable alternative for large-scale deployment. Radiative cooling inorganic multilayer films typically consist of a metallic reflective layer and dielectric layers with selective absorption properties in the atmospheric window [19]. The metallic reflective layer commonly employs materials with excellent solar reflectivity, particularly Ag and Al [20,21]. However, for large-scale applications, cost-effectiveness is crucial, making Al a more favorable alternative to Ag as the reflective layer. Most inorganic materials use periodic multilayer film structures with alternating high and low refractive indices to achieve daytime radiative cooling properties [1,4,22]. However, this approach results in a large number of film layers and thus increases the production cost. Furthermore, due to the weak bonding between Al and dielectric materials and the low solar reflectivity of Al, few studies have been conducted to design radiative cooling inorganic multilayers using Al as a metallic reflective layer [23]. Therefore, the design and fabrication of structurally simple, cost-effective inorganic multilayer films suitable for industrial production presents both significant challenges and promising development prospects in the field of radiative cooling. In this work, an innovative symmetric structure using Al as a reflector is proposed that achieves a remarkable net cooling power while significantly simplifying the conventional multilayer design. The resolution of the persistent interfacial adhesion problem between Al and Si_3_N_4_ layers through an optimized thermal treatment process. This study provides a reliable strategy for low-cost, high-performance inorganic multilayer films.

## 2. Materials and Methods

**Figure 1:**
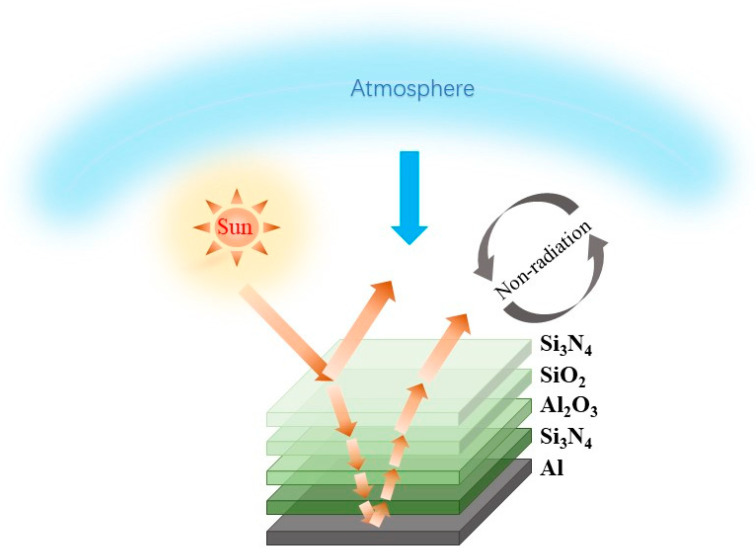
Schematic structure and heat flow diagram.

In this study, an inorganic multilayer film structure was designed: H-L-L-H, as shown in Figure 1. The inorganic multilayer films are prepared using JGP350C ultra-high vacuum multi-target magnetron sputtering (Sky Technology Development Co., Ltd. Chinese Academy of Sciences, Shenyang, China). A 2.5 × 2.5 cm^2^ glass substrate with a clean and scratch-free surface was selected prior to the deposition of the film, and it was successively soaked in alcohol and deionized water and ultrasonically cleaned for 15 min. The vacuum was evacuated to a background vacuum of 6 × 10^−4^ Pa before sputtering, and the air pressure in the chamber was adjusted to 0.5 Pa during sputtering. The Al layer was fabricated using a high-purity Al target (>99.999 wt%) through a direct current (DC) sputtering process [24]. The deposition was carried out with an argon flow rate of 40 standard cubic centimeters per minute (sccm), a DC power supply, and a sputtering power of 100 W. For the Si_3_N_4_ layer, a high-purity Si target (>99.99 wt%) was employed in a radio frequency (RF) sputtering process [25], with an Ar to N_2_ flow ratio of 50:10, an RF power supply, and a sputtering power of 200 W. The Al_2_O_3_ layer was deposited using an Al target (>99.999 wt%) with Ar (40 sccm) and O_2_ (6 sccm) as the sputtering and reactive gases, respectively, under an RF power of 200 W. Similarly, the SiO_2_ layer was prepared using a Si target (99.999 wt%) in a sputtering atmosphere of Ar (40 sccm) and O_2_ (4 sccm) with an RF sputtering power of 100 W. Due to the poor adhesion between the Al layer and the Si_3_N_4_ layer at room temperature, it is challenging to achieve a well-bonded multilayer structure. However, by heating the substrate to 450 °C during the deposition of the Si_3_N_4_ layer adjacent to the Al layer, an inorganic multilayer film with improved interfacial adhesion is successfully fabricated. Table S1 summarizes the deposition time and thicknesses of each layer.

The thickness of the layers was measured using a KLA-Tencor P7 surface profiler (KLA Corporation, Milpitas, CA, USA). Optical reflectance spectra were obtained in the wavelength ranges of 0.3–2.5 μm and 2.5–20 μm using a Lambda 950 UV-VIS-NIR spectrophotometer (Hitachi High-Tech Corporation, Tokyo, Japan) and a NICOLET 6700 smart FTIR spectrometer with an integrating sphere A562-G/Q (Thermo Fisher Scientific, Madison, WI, USA), respectively, with the latter measurements performed at an incidence angle of 8° using gold flakes as the diffuse reflectance standard. The phase composition of the films was analyzed by X-ray diffraction (XRD) using a Bruker D8 ADVANCE diffractometer (Bruker Corporation, Billerica, MA, USA) with Cu Kα radiation (λ = 1.541 Å) at a grazing incidence angle of 0.5°, with the X-ray tube operating at 40 kV and 40 mA. The cross-sectional morphology was examined using a ZEISS Gemini SEM 500 (Carl Zeiss AG, Oberkochen, Germany) high-resolution scanning electron microscope (HR-SEM). Additionally, the elemental composition of each layer was investigated using X-ray photoelectron spectroscopy (XPS) on a Thermo Scientific K-Alpha instrument (Thermo Fisher Scientific, Waltham, MA, USA). The adhesion strength of the thin films was evaluated using a micro-scratch tester of Anton Par NHT + MCT (Anton Paar GmbH, Graz, Austria). The tests were performed under the following conditions: a loading rate of 29.97 N/min, a scanning load of 0.03 N, a scanning speed of 3 mm/min, and a scanning length of 3 mm. Data were acquired at a sampling rate of 30 Hz, and a diamond indenter was used for the measurements.

## 3. Results and Discussion

### 3.1. Structural Characterization

The notable cost advantage of Al over Ag as a reflective layer material is evident in both raw material expenses and processing requirements. While this study employs a minimal 200 nm reflective layer, industrial-scale deployment would require material volumes thousands to tens of thousands of times greater, making cost considerations paramount. In this study, pure metal targets were employed for sample fabrication. Al targets offer exceptional cost-effectiveness, with the prices remaining stable at just USD4–7 (≥99.9% purity) compared to Ag target’s precious metal market volatility (USD200–500, ≥99.9% purity). Even at high purity levels (≥99.99%), Al maintains a dramatic price advantage, costing merely a fraction (less than 1%) of equivalent-grade Ag targets. This substantial price differential makes it a clearly superior choice for cost-sensitive applications. In addition, the simplicity of the material structure proposed in this manuscript results in two main cost reductions: Firstly, the proposed structure requires only four layers (excluding the reflective layer), largely reducing the costs of materials in industrial-scale fabrication. For instance, this design achieves a 67% reduction in layer count compared to the conventional 12-layer film stack (excluding the reflective layer) reported in Ref. [26]. Secondly, this simple structure saves production process costs and time.

**Figure 2:**
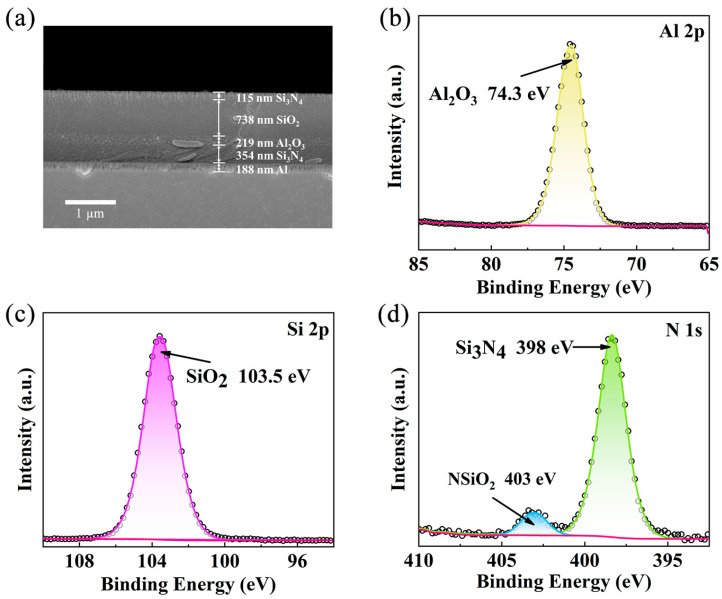
(**a**) Scanning electron micrographs (SEM) of cross sections of the multilayers; X-ray photoelectron spectra (XPS) of (**b**) Al 2p, (**c**) Si 2p, and (**d**) N 1s.

**Figure 3:**
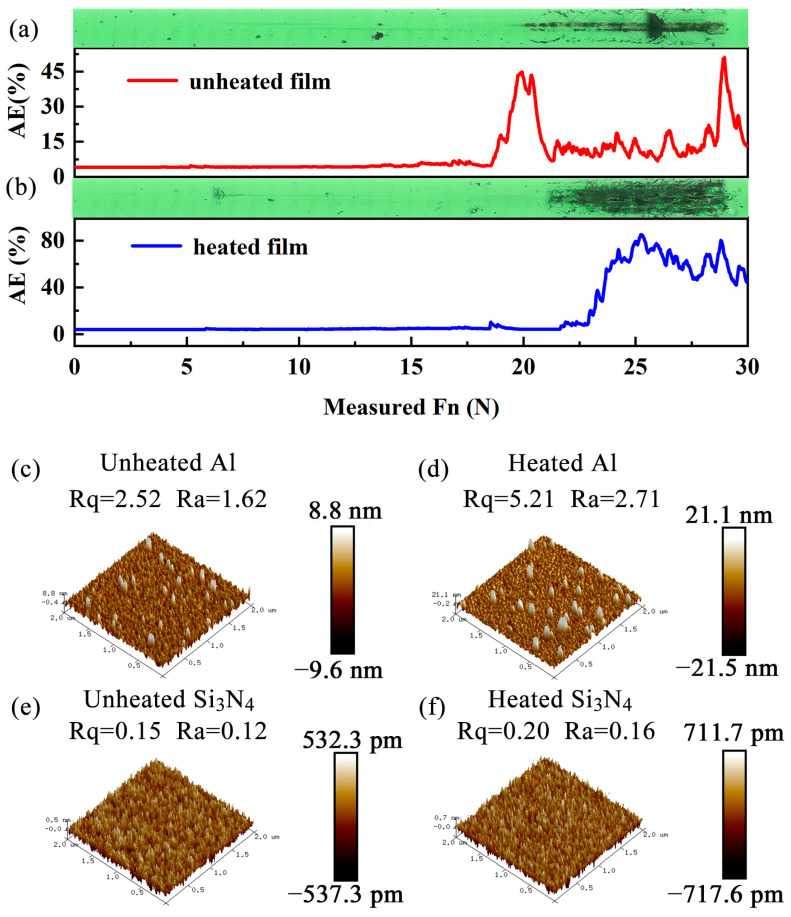
Scratch meter testing of films prepared (**a**) with and (**b**) without substrate heating. Three-dimensional (3D) images scanned by atomic force microscope (AFM): (**c**) Al layer prepared without heating substrate; (**d**) Al layer prepared without heating substrate and then heated at 450 °C for 55.73 min (the conditions are the same as when preparing the whole layer); (**e**) Si_3_N_4_ prepared without heating substrate; (**f**) Si_3_N_4_ prepared by heating the substrate at 450 °C.

Figure 2a presents a cross-sectional scanning electron microscopy (SEM) image of the inorganic multilayer film. The SEM image reveals distinct interfaces between the layers. The interface between Al and Si_3_N_4_ is well adhesion. Combining the results of SEM characterization with those of scratch meter testing (Figure 3a,b) shows that the Al-Si_3_N_4_ interface is well bonded. The total thickness of the multilayer film, obtained by summing the individual layer thicknesses from the SEM analysis, is approximately 1.6 μm. The small thickness and low production cost of this material make it highly suitable for commercial applications [27,28]. To characterize the physical phases of each material in the multilayer film, XRD analysis on the entire film is conducted [29,30], as shown in Figure S1. The presence of only Al crystallization peaks in the XRD results indicates that Si_3_N_4_, Al_2_O_3_, and SiO_2_ are amorphous. To further investigate the composition, the single-layer films of Al_2_O_3_, Si_3_N_4_, and SiO_2_ on Si substrates were fabricated and analyzed using XPS, as presented in Figure 2b–d. To minimize potential oxidation from air, the samples were etched down by 30 nm prior to XPS characterization. The Al 2p spectrum in Figure 2b shows a single peak at 74.3 eV, corresponding to Al_2_O_3_. In Figure 2c, the Si 2p spectrum exhibits a peak at 103.5 eV, indicative of SiO_2_. Similarly, the N 1s spectrum in Figure 2d displays two peaks at 398 eV and 403 eV, which are attributed to Si_3_N_4_ and NSiO_2_, respectively. The presence of NSiO_2_ suggests some oxidation resulting from either insufficient vacuum conditions in the sputtering chamber or brief air exposure during sample transfer for testing. Nevertheless, the XPS characterization confirms the successful formation of Si_3_N_4_ in the fabricated samples.

During the fabrication process, the surface of the inorganic multilayer film cracked, leading to sample failure. Through systematic investigation, the phenomenon was primarily caused by poor adhesion between the Al and Si_3_N_4_ layers. After extensive experimentation, the cracking issue was effectively mitigated by implementing substrate heating during the deposition of the Si_3_N_4_ layer adjacent to the Al layer. Specifically, when depositing the Si_3_N_4_ layer in direct contact with the Al layer, the substrate temperature was maintained at 450 °C for 55.73 min to ensure optimal film adhesion. The adhesion strength was subsequently evaluated using a micrometer-scratch tester, as illustrated in Figure 3a,b. The results depicted in the figure demonstrate that the Al/Si_3_N_4_ layer fabricated with substrate heating exhibits superior adhesion strength compared to its counterpart prepared without substrate heating. The improved interfacial adhesion was further confirmed through tape peel tests, which demonstrated excellent bonding strength in the fabricated samples, as shown in Figure S2 [31,32,33].

In order to further study the internal mechanism of the effect of the heated substrate on the bonding force of the multilayer film, the roughness changes of Al and Si_3_N_4_ before and after heating were tested, as shown in Figure 3c–f. Figure 3c–f reveals that the Al layer deposited without substrate heating exhibits a smoother surface compared to its heated counterpart. In contrast, the Si_3_N_4_ layers deposited with and without heating demonstrate comparable surface flatness. This phenomenon suggests that the heating treatment primarily enhances the surface roughness of the Al layer, thereby improving the interfacial adhesion between the Al and Si_3_N_4_ layers. Figure S3 and Table S2 confirm that substrate heating effectively addresses the adhesion issue between the metallic Al layer and the dielectric Si_3_N_4_ layer without compromising the optical properties of Si_3_N_4_. These findings provide valuable insights into the role of substrate heating in optimizing the structural integrity of multilayer films through surface modification [34].

### 3.2. Optical Properties

**Figure 4:**
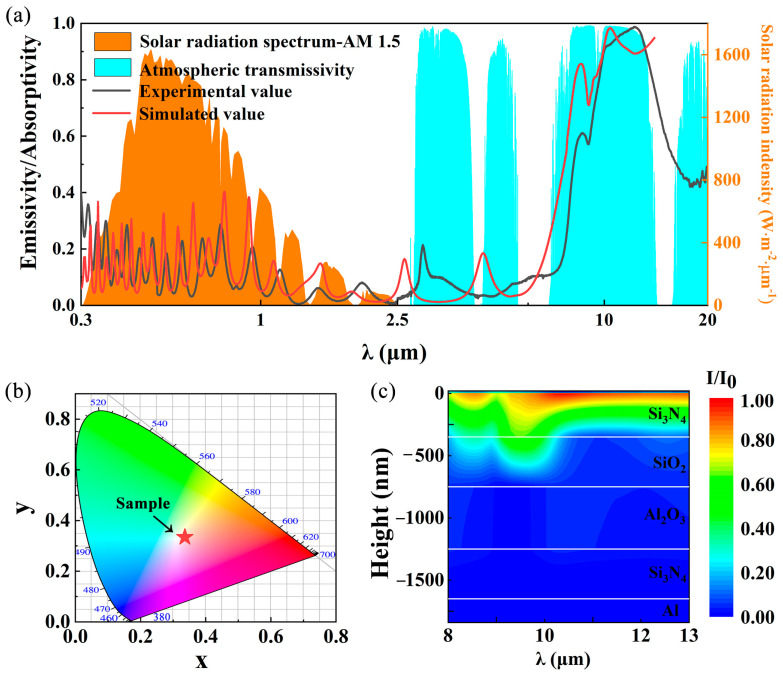
(**a**) The calculated and experimental emission/absorption spectra, (**b**) chromaticity diagram, and (**c**) normalized energy flow density of the inorganic multilayer film.

The experimental and simulated emission/absorption spectra of the inorganic multilayer film in the wavelength range of 0.3–20 μm are presented in Figure 4a. Figure 4a shows the spectrum with solar irradiance AM1.5 and atmospheric transmittance as the background [35]. The significant discrepancy between the simulation results and the experimental measurements is mainly due to the following two reasons: (1) due to the difficulty of actually measuring the optical constants in the infrared band, we used the optical constants from the Palik manual in simulating the multilayer film spectra, which slightly differ from the optical constants of the actual prepared samples; (2) there is a certain difference between the thickness of the actual multilayer film and the simulated thickness. The experimental data exhibit a discontinuity at 2.5 μm, primarily attributed to the transition between different measurement instruments used for various wavelength ranges. The multilayer film demonstrates excellent solar reflectance, with oscillatory peaks in the solar spectrum (0.3–2.5 μm) resulting from optical interference at the SiO_2_/Si_3_N_4_, SiO_2_/Al_2_O_3_, and Si_3_N_4_/Al_2_O_3_ interfaces. According to the literature [36], within the 0.3–2.5 μm range, the real part of the refractive index (n) is approximately 1.5 for SiO_2_, 1.8 for Al_2_O_3_, and 2.5 for Si_3_N_4_. The H-L-L-H configuration establishes a graded refractive index transition at the air-coating interface (n(air) = 1 → n(Si_3_N_4_) = 2.5 → n(SiO_2_) = 1.5 → n(Al_2_O_3_) = 1.8 → n(Si_3_N_4_) = 2.5), which effectively minimizes Fresnel reflection losses compared to the H-L-H-L structure. By reducing parasitic solar absorption and enhancing broadband reflection, this design improves the radiative cooling performance of the multilayer film, as it allows more efficient thermal emission in the atmospheric transparency window (8–13 μm) while maintaining high solar reflectivity (0.3–2.5 μm). The multilayer film exhibits high selective emission in the atmospheric window (8–13 μm), primarily due to the synergistic compensation of intrinsic absorption peaks from SiO_2_, Al_2_O_3_, and Si_3_N_4_ within this range. Additionally, an absorption peak appears near 3–5 μm, caused by photon–phonon resonance in the materials. This spectral feature is particularly beneficial for radiative cooling performance as it coincides with a narrow atmospheric window in this wavelength range [37]. Compared to existing Al-based reflective inorganic multilayer films, this work demonstrates superior spectral characteristics while featuring a simpler structure and lower production cost, making it more suitable for industrial-scale manufacturing.

Figure 4b presents the chromaticity diagram calculated from the reflectance spectrum of the multilayer film. The color coordinates of the film are (0.336, 0.334), which closely resemble those of pure white (0.333, 0.333) [38]. This similarity is primarily attributed to the high reflectance of the inorganic multilayer film across the solar spectrum. As shown in Figure 4c, the normalized energy flux density (I/I_0_) distribution through the film depth was simulated using the transfer matrix method. It can be observed that the energy flux density of the multilayer film is mainly concentrated in the Si_3_N_4_ and SiO_2_ layers, which indicates that the inorganic multilayer film structure restricts the light in Si_3_N_4_ and SiO_2_ through interference and is further absorbed. The analysis further demonstrates that SiO_2_ exhibits two intrinsic absorption peaks in the 8–10 μm range, while the majority of light is absorbed by the surface Si_3_N_4_ layer.

Table 1 presents the radiative cooling performance parameters calculated by incorporating the experimentally measured reflectance spectrum of the multilayer film into MATLAB (R2016a) simulations. The inorganic multilayer film demonstrates a solar reflectance of 89.57% in the solar spectrum and an emissivity of 83.41% within the atmospheric window. Under ideal conditions, the film achieves a net cooling power of 63.38 W·m^−2^, with a temperature of 6 K below ambient when the non-radiative heat transfer coefficient is 6.9. These results highlight the film’s exceptional radiative cooling capabilities and its potential for effective thermal management applications.

### 3.3. Radiative Cooling Properties

**Figure 5:**
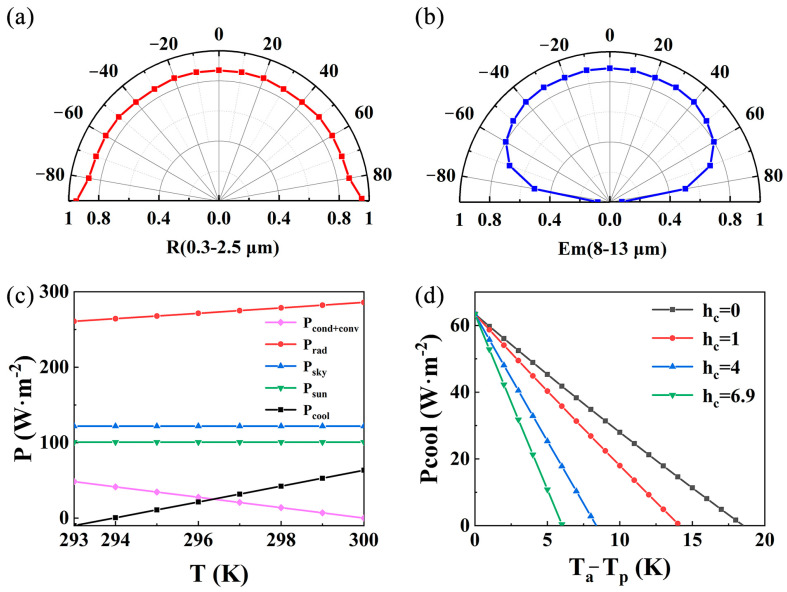
(**a**) The reflectivity of sunlight at different incidence angles; (**b**) Atmospheric window emissivity at different incidence angles; (**c**) Curves of each thermal power and (**d**) net cooling power with temperature. In (**c**,**d**), P_cond+conv_ is non-radiation power; P_rad_ is the radiation power; P_sky_ is the power of absorption from the atmosphere; P_sun_ is the power of absorption from sunlight; P_cool_ is the net cooling power; The h_c_ is non-radiative heat transfer coefficient; T_a_ is the temperature of ambient; T_p_ is the temperature of the sample.

Figure 5a illustrates the calculated solar reflectance, with the polar plot demonstrating minimal variation in the film’s solar reflectance across different angles [39]. Figure 5b presents the calculated emissivity within the atmospheric window (8–13 μm), revealing stable performance at incident angles below 60° and a significant decrease at angles above 60° [40]. This angular stability of spectral performance across a wide range of incident angles aligns well with practical atmospheric conditions, as the atmosphere is most transparent at the zenith and least transparent near the horizon [41,42].

The maximum temperature difference between the multilayer film and the ambient environment occurs when the net cooling power (P_cool_) reaches zero [43]. To further analyze the relationship between various power densities and temperature, the thermal power curves as a function of temperature at h_c_ = 6.9 were calculated [44], as shown in Figure 5c. As the film temperature increases from 293 K to 300 K, P_cool_ rises from approximately −10 W·m^−2^ to 63 W·m^−2^, indicating a transition from heat absorption to a cooling state, which demonstrates the strong temperature dependence of P_cool_. The cooling effect reaches its maximum when the temperature drops to 294 K, where P_cool_ becomes zero. The figure also reveals that the absorbed solar radiation power (P_sun_) and atmospheric radiation power (P_sky_) remain constant regardless of the film temperature. The radiative power (P_rad_) from the multilayer film increases with temperature, ranging from 261 W·m^−2^ to 282 W·m^−2^, while the non-radiative heat exchange power (P_cond+conv_) decreases as temperature increases.

Figure 5d illustrates that the intersection point between the curve and the horizontal axis represents the maximum cooling temperature, where the net cooling power is zero. As the non-radiative heat transfer coefficient increases, the maximum temperature difference between the film and the environment decreases. Similarly, for a given temperature difference, the net cooling power decreases with increasing non-radiative heat transfer coefficient. Conversely, at a fixed non-radiative heat transfer coefficient, the net cooling power decreases as the temperature difference increases. Under ideal conditions with zero non-radiative heat transfer (h_c_ = 0), the maximum temperature reduction reaches 18 K. This potential cooling performance decreases to 14 K at h_c_ = 1, 8 K at h_c_ = 4, and 6 K at h_c_ = 6.9.

**Figure 6:**
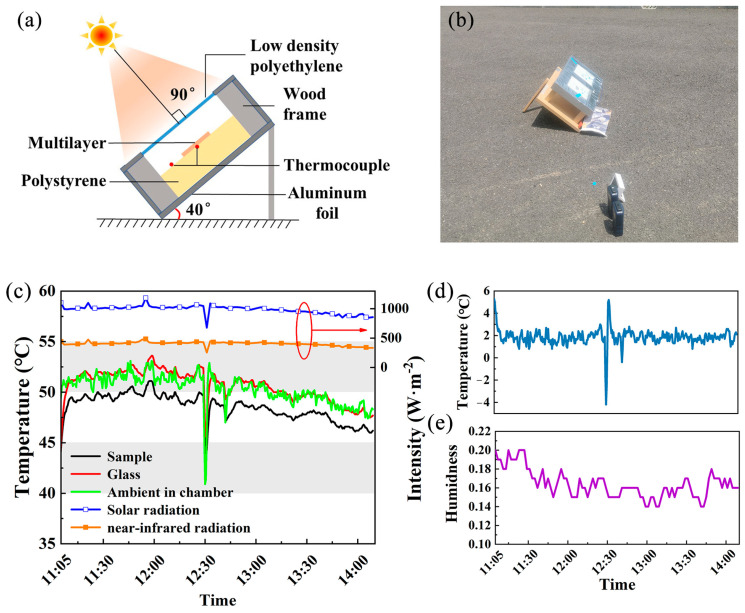
Outdoor temperature measurement experiment. (**a**) Schematic diagram and (**b**) on-site photos; (**c**) temperature curve over time; (**d**) the temperature difference between the outdoor temperature test sample and the glass of the comparison sample; (**e**) real-time humidity data of outdoor temperature measurement experiment.

Figure 6a illustrates the schematic diagram of the outdoor temperature measurement experiment. The outdoor temperature measurement methods used in this study are the same as those mentioned in previous articles [36]. The outdoor temperature measurement experiment was conducted on 20 May 2024, in Beijing (116° E, 40° N), with Figure 6b showing the experimental site photograph [45]. Temperature measurements were taken using K-type thermocouples connected to the bottom of the multilayer film and inside the chamber, as shown in Figure 6a. Since the multilayer film is deposited on a glass substrate, we conducted temperature measurements using bare glass as the control group to isolate the substrate’s influence. The experimental and control samples differ solely in the presence or absence of the inorganic multilayer film. Therefore, the sample and control sample have almost the same thickness and size. The temperature measurement experiment spanned 3 h and 34 min, from 10:35 a.m. to 2:09 p.m. As shown in Figure 6c, the multilayer film demonstrated a maximum temperature reduction of approximately 3 °C compared to the glass reference sample. Figure 6d reveals that the minimum temperature reduction of the inorganic multilayer film occurred at noon when solar radiation was strongest, confirming that solar radiation attenuates the radiative cooling effect of the inorganic multilayer film. Real-time humidity data from the China Meteorological Administration (CMA) [46] were used to plot the humidity curve, presented in Figure 6e. It should be noted that discrepancies between actual temperature measurements and ideal simulation values arise from factors such as atmospheric transmittance, humidity, solar irradiance, substrate thermal conductivity, and radiation from nearby buildings and vegetation. The inorganic multilayer film demonstrates significant sub-ambient cooling performance, which holds considerable importance for daytime radiative cooling applications.

## 4. Conclusions

In this study, a cost-effective inorganic multilayer film with a simple structure (Si_3_N_4_/SiO_2_/Al_2_O_3_/Si_3_N_4_/Al) was designed and optimized. SEM, XRD, and XPS characterization tests confirmed that the parameters such as composition and thickness of the prepared inorganic multilayer films were consistent with the designed ones. The interfacial adhesion between the Al metal layer and Si_3_N_4_ was significantly enhanced through heat treatments during deposition. The study shows that the improved adhesion primarily resulted from increased surface roughness of the Al layer. The optimized multilayer film demonstrates excellent radiative cooling properties, with an emissivity of 83.41% in the atmospheric window and a solar reflectance of 89.57% across the solar spectrum. Under direct sunlight, the multilayer film achieved significant sub-ambient cooling performance. These results provide an effective approach for daytime radiative cooling and offer a reliable strategy for developing commercially viable radiative cooling inorganic materials.

## Figures and Tables

**Table 1:** Radiative cooling parameters of inorganic multilayer films.

R (0.3~2.5 μm) (%)	Εm (8~13 μm) (%)	P_net-cooling_ (W·m^−2^)	P_rad_ (W·m^−2^)	P_sky_ (W·m^−2^)	P_sun_ (W·m^−2^)	P_cond+conv_ (W·m^−2^)	ΔT(h = 6.9) (K)
89.57	83.41	63.38	285.88	121.81	100.69	0	6.00

## Data Availability

The original contributions presented in this study are included in the article/Supplementary Material. Further inquiries can be directed to the corresponding authors.

## Supplementary Materials

The following supporting information can be downloaded at: https://www.mdpi.com/article/10.3390/ma18081729/s1. Table S1: Thickness and deposition time of each film layer; Table S2: Solar reflectance and atmospheric window emissivity of inorganic multilayers; Figure S1: X-ray diffraction (XRD) spectrum. Figure S2: Tape peeling experiments. (a,d) are photographs of the sample before peeling; (b) the process of peeling the surface of the sample with polyimide (PI) tape; (c) the sample was still intact when peeled 20 times with PI tape; (e) the process of peeling the surface of the sample with commercial 3M tape; (f) the sample was still intact when peeled 20 times with 3M tape; Figure S3: (a) Optical constants of Si_3_N_4_ prepared under substrate-heated and unheated conditions in the solar band; (b) reflectance and transmittance spectra of Si_3_N_4_ prepared under substrate-heated and unheated conditions in the solar band; emission/absorption spectra of inorganic multilayers prepared at different substrate-heated temperatures in the (c) solar band and (d) mid-infrared band.

## Abbreviations

The following abbreviations are used in this manuscript:

H	High refractive indices
L	Low refractive indices
DC	Direct current
RF	Radio frequency
sccm	Standard cubic centimeters per minute
I/I_0_	Normalized energy flux density
AM1.5	Air mass 1.5
