# Supplementary material for: Cost-Effective Inorganic Multilayer Film for High-Performance Daytime Radiative Cooling
Source: Materials (Basel). 2025 Apr 10;18(8):1729. doi: 10.3390/ma18081729 (PMC12028864; doi:10.3390/ma18081729)
Supplement: Supplementary file 1 [file materials-18-01729-s001.zip › materials-3550218-supplementary.pdf]

Cost-Effective Inorganic Multilayer Film for High-Performance Daytime Radiative Cooling

Huan Liu, Yingxin Yang, Atsha Ambar, Zhiqiang Fan, Ying Sun*, Cong Wang*

S1 Thickness and deposition time of each film layer

Table S1. Thickness and deposition time of each film layer.

Materials	Si_3N_4	Si_3N_4	SiO_2	Al_2O_3	Al
Thickness [nm]	354	115	738	219	188
Deposition time [min]	55.73	48.77	93.23	143.68	1.00

Table S1 summarizes the thickness and deposition time for each layer in the multilayer film structure.

S2 Characterization

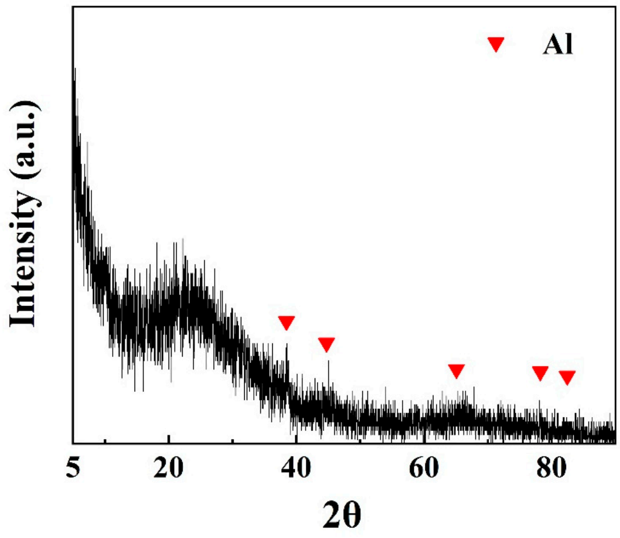

Figure S1. X-ray diffraction (XRD) spectrum

To characterize the physical phases of each material in the multilayer film, X-ray diffraction (XRD) analysis on the entire film is conducted [28, 29], as shown in Figure S1. The presence of only Al crystallization peaks in the XRD results indicates that Si_3N_4,

Al_2O_3, and SiO_2 are amorphous. The broad hump observed between 20° and 30° originates from the amorphous glass substrate.

S3 Interface adhesion

We conducted tape peel tests to demonstrate the excellent interfacial adhesion of our fabricated samples, as shown in Figure S2. Figures S2(a) and (d) display the samples before the peel test, while Figure S2(b) illustrates the process of attaching polyimide (PI) tape to the sample surface. Figure S2(c) shows that the sample remains intact after 20 peel cycles with PI tape. Similarly, Figure S2(e) depicts the application of commercial 3M tape to the sample surface, and Figure S2(f) confirms that the sample withstands 20 peel cycles with 3M tape without damage. These results provide strong evidence that we have successfully fabricated inorganic multilayer film samples with superior interfacial adhesion, effectively addressing the bonding issue between the Al layer and Si_3N_4.

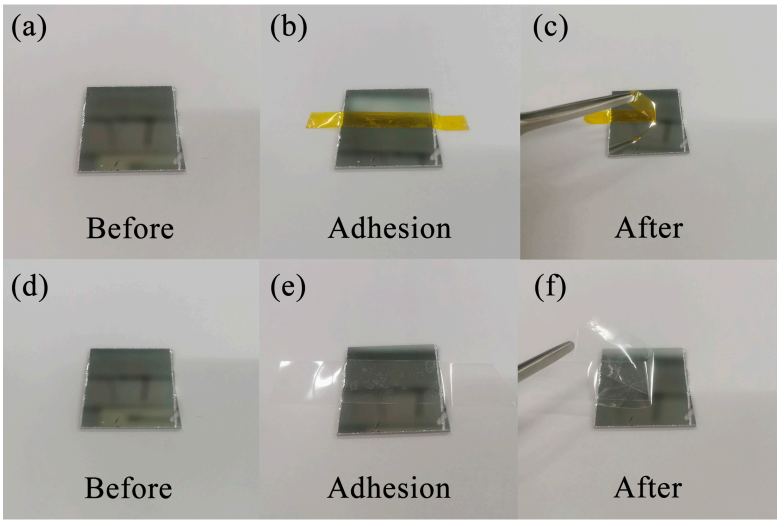

Figure S2. Tape peeling experiments. (a) and (d) are photographs of the sample before peeling; (b) the process of peeling the surface of the sample with polyimide (PI) tape; (c) the sample was still intact when peeled 20 times with PI tape; (e) the process of peeling the surface of the sample with commercial 3M tape; (f) the sample was still intact when peeled 20 times with 3M tape.

S4 Effect of substrate heating on the optical properties of inorganic multilayer films

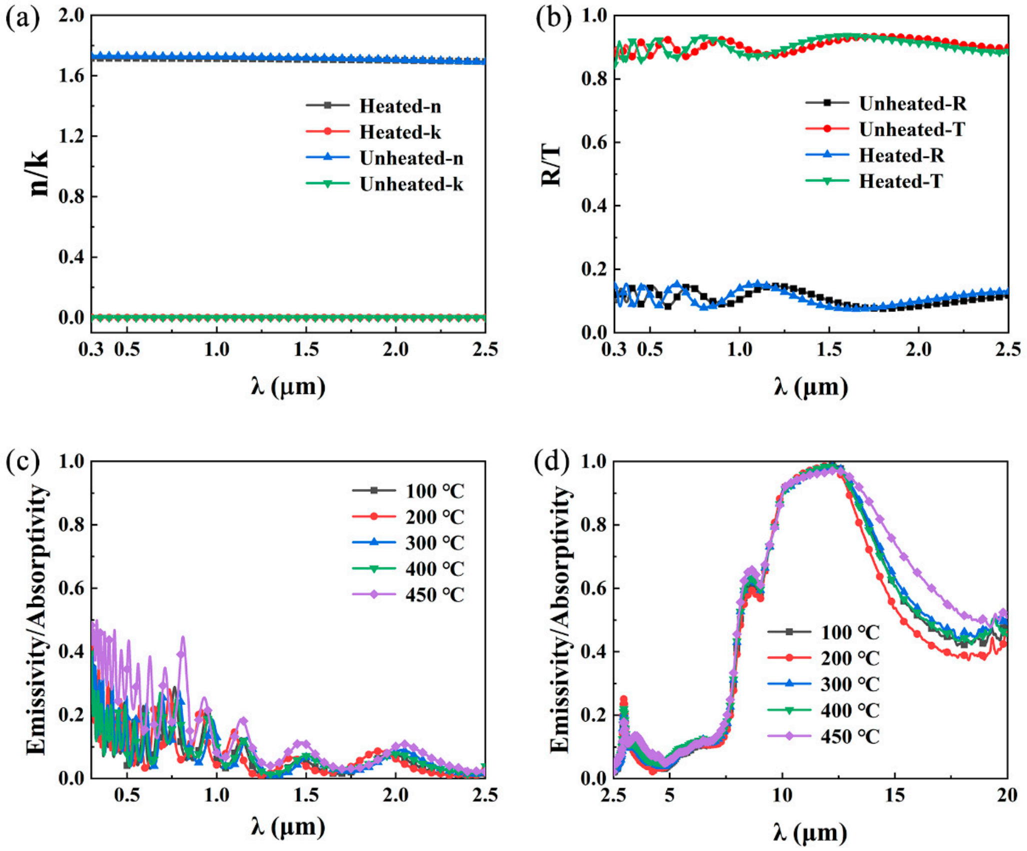

Figure S3. (a) Optical constants of Si_3N_4 prepared under substrate-heated and unheated conditions in the solar band; (b) Reflectance and transmittance spectra of Si_3N_4 prepared under substrate-heated and unheated conditions in the solar band; Emission/absorption spectra of inorganic multilayers prepared at different substrate-heated temperatures in the (c) solar band and (d) mid-infrared band

To further confirm the negligible effect of heating on the optical properties of Si_3N_4, we fitted the reflection and transmission spectra using SCOUT software, employing the Brendel oscillator and Tauc-Lorentz oscillator models. The derived optical constants for Si_3N_4 under both heated and unheated conditions are presented in Figure S3(a), showing no significant differences, which further supports the conclusion that heating has minimal

impact on the optical performance of Si_3N_4.

To demonstrate that substrate heating during the preparation of Si_3N_4 has minimal impact on the optical properties of the inorganic multilayer film, we first measured the transmission and reflection spectra of Si_3N_4 deposited with and without substrate heating, as shown in Figure S3(b). The spectra reveal that the optical properties of Si_3N_4 remain largely unchanged regardless of heating, although slight differences in interference fringes are observed. These variations arise from minor discrepancies in deposition rates caused by differences in temperature and target-to-substrate distance during the two preparation processes, leading to small variations in film thickness and thus shifts in the interference fringe positions.

Subsequently, we prepared inorganic multilayer films at different substrate heating temperatures and measured their emission/absorption spectra, as shown in Figures S3(c)-(d). The results indicate that the emission/absorption spectra of the multilayer film in both the solar and mid-infrared regions are largely unaffected by temperature variations. Due to equipment limitations, the maximum heating temperature tested was 450 °C. These findings suggest that the optical performance of the inorganic multilayer film remains consistent across a substrate heating temperature range of 100 °C to 450 °C.

Table S2 presents the calculated solar reflectance and atmospheric window emissivity of the inorganic multilayer film at different Si_3N_4 layer heating temperatures. The data further demonstrate that the spectral performance of the multilayer film remains largely unaffected within a certain range of substrate temperatures. To verify the strong interfacial adhesion across these temperatures, we conducted tape peel tests on samples prepared at the other four temperatures, obtaining results consistent with those at 450 °C. These findings confirm that substrate heating effectively addresses the adhesion issue between the metallic Al layer and the dielectric Si_3N_4 layer without compromising the optical properties of Si_3N_4. This approach provides a reliable solution for enhancing

interfacial bonding while maintaining optimal optical performance in the multilayer film structure.

Table S2. Solar reflectance and atmospheric window emissivity of inorganic multilayers prepared at different substrate heating temperatures.

Temperature	R(0.3~2.5 μm)	Em(8~13 μm)
100 ℃	89.6	83.4
200 ℃	89.2	83.1
300 ℃	88.5	83.8
400 ℃	88.9	83.9
450 ℃	78.3	84.3
